# Laryngeal Abscess: A 34-Year Review

**DOI:** 10.1007/s12070-025-05323-9

**Published:** 2025-01-18

**Authors:** Aida Veiga-alonso, Ramón Cobo-Díaz, Belén Salvatierra-Vicario, Patricia Corriols-Noval, Carmelo Morales-Angulo

**Affiliations:** 1 Department of Otolaryngology, Marqués de Valdecilla University Hospital, Santander, Spain; 2 Institute of Research Valdecilla (IDIVAL), Santander, Spain; 3 Department of Medical and Surgical Sciences, University of Cantabria, Santander, Spain; 4 Department of Otolaryngoloy, Hospital Universitario Marqués de Valdecilla, Av Valdecilla s/n, 39011 Santander, Spain

**Keywords:** Abscess, Laryngeal, Tracheostomy, Epidemiology

## Abstract

This study aimed to investigate the frequency, clinical-epidemiological characteristics, and management of laryngeal abscesses in our hospital setting. We included all patients treated for primary laryngeal abscesses in the Otorhinolaryngology Department of a tertiary hospital over a 34-year period. Patients with abscesses believed to have originated from adjacent regions were excluded. Thirteen patients with laryngeal abscesses were identified, with epiglottitis being the most common etiology (61%). Three patients were immunosuppressed. Four patients required urgent tracheotomy, and one underwent cricothyrotomy due to upper airway obstruction. All cases were drained under general anesthesia via a transoral route. Five patients required admission to the intensive care unit, and two experienced severe complications. There were no fatalities. Laryngeal abscesses are rare, with epiglottitis being the predominant cause. These cases pose a high risk of upper airway obstruction, highlighting the importance of early diagnosis and prompt treatment by a multidisciplinary team to prevent complications.

## Introduction

Deep head and neck abscesses, including those involving the larynx, necessitate timely intervention. Laryngeal abscesses may arise as primary lesions within the larynx or secondary to extension from adjacent areas. Various etiologies, such as epiglottitis, laryngopyocele, foreign body impaction, caustic ingestion, or iatrogenic factors, can precipitate primary laryngeal abscess formation [1–3].

Certain risk factors, including poorly controlled diabetes, HIV infection, autoimmune disorders, or immunosuppressive therapy, may predispose individuals to laryngeal abscess development, although spontaneous occurrences are also observed [2, 4].

Given the potential for rapid upper airway compromise in laryngeal abscess cases, prompt diagnosis and intervention are paramount. Despite the significance of this condition, literature on laryngeal abscess series remains scarce, primarily comprising descriptive clinical [1]. Therefore, we aimed to analyze cases treated over an extended period in our institution to better understand the clinical-epidemiological profile and management strategies employed.

## Methods

We conducted a retrospective analysis of patients treated for primary laryngeal abscesses at Marqués de Valdecilla University Hospital, a tertiary facility serving a catchment area of 560,000 individuals, from January 1, 1990, to December 31, 2023.

Patients presenting with suspected laryngeal abscesses that were suspected to have originated from adjacent regions, such as the retropharynx, peritonsillar area, floor of the mouth, or parapharyngeal area, and subsequently spread to the larynx, were excluded from the study.

Data on patient demographics, abscess etiology, risk factors, clinical presentation, imaging findings, culture results, airway interventions, treatments, and outcomes were collected and analyzed using SPSS software (SPSS 19.0 for Windows).

The authors assert that all procedures contributing to this work comply with the ethical standards of the relevant national and institutional guidelines on human experimentation (Ethical Committee for Research with Medicines and Health Products of Cantabria: code 2023.383, 10/11/2023) and with the Helsinki Declaration of 1975, as revised in 2008.

## Results

Over the study period, thirteen patients ranging in age from 44 to 96 years were treated for laryngeal abscesses in our department, accounting for 0.4% of all deep head and neck abscess cases. Table 1 summarizes the main patient data.

**Table 1:** Summary of the clinical-epidemiological characteristics of our series of patients

Nº/Age/sex	Etiology/Localization	Risk factors	Clinical presentation	Imaging study	Airway management	Treatment	Microorganism
1/56/Female	Epiglottic cyst	No	Odynophagia/ dyspnoea/Inspiratory stridor	No	Urgent tracheostomy	Transoral drainage	*Streptococcus pneumoniae*
2/62/Female	Aritenoepiglottic cyst	No	Fever/odynophagia	Yes (CT)	Intubation with an endoscope (awake patient)	Transoral drainage	ND
3/82/Male	Laringocele	No	Dyspnoea/inspiratory stridor	No	Urgent tracheostomy	Transoral drainage	Mixed bacterial growth with Gram-positive rods
4/81/Male	Laringocele	Diabetes type II. Previous fat injection	Fever/dysphonia	Yes (CT)	Intubation with an endoscope (awake patient)	Transoral drainage	ND
5/61/Female	Foreign body/cricoid	No	Odynophagia	Yes (CT)	No	Transoral drainage	*Streptococcus neumoniae*
6/58/Male	Epiglottitis	No	Fever/Odynophagia/dyspnoea/inspiratory stridor	No	Tracheostomy	Transoral drainage	*Streptococcus pneumoniae*
7/67/Male	Epiglottitis	Inmunosupression	Fever/odynophagia/ dyspnoea	Yes (CT)	Intubation with videolaryngoscopy (awake patient)	Transoral drainage	*Staphylococcus aureus*
8/59/Male	Epiglottitis	No	Odynophagia	Yes (CT)	Intubation with videolaryngoscopy (awake patient)	Transoral drainage	*Streptoccccus group B*
9/96/Male	Epiglottitis	No	Fever/ odynophagia/dyspnoea/inspiratory stridor	No	Cricothyrodomy	Transoral drainage	ND
10/57/Male	Epiglottitis	No	Oodynophagia/dyspnoea	Yes (CT)	Intubation with an endoscope (awake patient)	Transoral drainage	*Streptococcus Bhemolítico*
11/44/Male	Epiglottitis	Diabetes type II	Odynophagia/dyspnoea/inspiratory stridor	Yes (CT)	Tracheostomy	Transoral drainage	ND
12/71/Female	Epiglottitis	Immunosupressor treatment for reumatoid arthritis	Odynophagia	Yes (CT)	Videolaryngoscopy	Transoral drainage	*Streptococcus Milleri*
13/82/Male	Epiglottitis	No	Odynophagia	Yes (CT)	Videolaryngoscopy	Transoral drainage	Mix bacterial grow

Epiglottitis was identified as the most common cause, observed in eight patients (61%). Among the patients, three (23%) had identifiable risk factors, with one suffering from poorly controlled type II diabetes and the other two presenting immunosuppression conditions.

All patients presented with sore throat and vocal disturbances, with seven (54%) experiencing dyspnea and inspiratory stridor, severe in five cases. Four patients required urgent tracheostomy, while one underwent emergent cricothyrotomy due to upper airway compromise. Computed tomography was performed prior to surgical intervention in most cases, with subsequent intubation performed in five patients using video laryngoscopy or nasofibroscopy while awake in the operating room.

Drainage of the abscess was performed transorally under general anesthesia in all cases, with Streptococcus pneumoniae being the most frequently isolated microorganism in abscess cultures.

Hospital admission ranged from 3 to 14 days, with a median of 8. Five patients required intensive care unit admission, and two patients experienced severe complications such as septic shock and acute myocardial infarction with bilateral pleural effusion. There were no fatalities, and no reintervention was necessary. Patients who underwent tracheostomy were decannulated after 2 to 7 days.

## Discussion

Laryngeal abscesses are exceedingly rare, accounting for less than 1% of cases of head and neck abscesses (0.4% in our series). Although the epiglottis is the most common site, they can occur in any area of the larynx (Table 2).

**Table 2:** Classification of laryngeal abscesses

1. Primary laryngeal abscess	
	a. Thyroid cartilage [5–7]
	b. Cricoid/retrocricoid [2, 8]
	c. Epiglottic [9]
	d. Laryngeal ventricle [10]
	e. Cricoarytenoid joint [11]
	f. Various areas of the larynx involved [12]
2. Secondary laryngeal abscesses (by extension to the larynx from abscesses located in other areas of the neck)	

The typical clinical presentation includes sore throat (100%) and voice changes (100%), often progressing rapidly to odynophagia and, in some cases, dyspnea and inspiratory stridor (7 out of 13 in our series). Associated febrile conditions are not uncommon. Endoscopic examination is crucial for accurate diagnosis, with findings such as edema, erythema, glottic stenosis, and even vocal cord hypomotility frequently observed in adults [1]. Although our study did not include pediatric cases, suspicion of a laryngeal abscess in children warrants caution to avoid manipulation outside of controlled environments like an intensive care unit or an operating room due to the risk of acute laryngeal closure.

Risk factors for laryngeal abscess development are similar to those for other head and neck abscesses, including poorly controlled diabetes or immunosuppressive treatments, present in just under a third of our patients. However, they can also occur spontaneously without identifiable causes [13]. Various factors such as external trauma [14], iatrogenic causes, foreign bodies, or tumors can contribute to laryngeal abscess formation [2, 15, 16]. Table 3 summarizes the most frequent causes of laryngeal abscesses described in the literature. Our series identified a foreign body, superinfection of a laryngeal cyst, laryngocele, and, notably, epiglottitis as causal factors.

**Table 3:** Causes of laryngeal abscesses

1. Epiglottitis [9]				
2. Trauma				
	A. External [14]			
	B. Internal:			
		Iatrogenic to:		
			a. Urgent orotracheal intubation [2]	
			b. Prolonged intubation [7, 8]	
			c. Placement of nasogastric tube [2]	
			d. Injection laryngoscoplasty with:	
				Hyaluronic acid [16]
				Micronized Dermis [17]
			e. Negative pressure wound therapy [15]	
			f. Laryngeal laser surgery for laryngeal carcinoma [5]	
		Non-iatrogenic		
			Foreign body impaction (our series)	
3. Laryngeal cysts (our series)				
4. Superinfection of laryngopiocele [10]				
5. Radiotherapy [2, 18]				
6. Systemic infectious diseases: tuberculosis [19]				
7. Spontaneous [13]				
8. Cricoarteroid arthritis due to RA [11]				
9. Relapsing current polychondritis [7]				

Historically, laryngeal abscesses were primarily caused by systemic diseases such as typhoid fever, tuberculosis, syphilis, or measles, but such cases are now rare [1, 3]. Secondary laryngeal abscesses due to common microorganisms seen in other head and neck abscesses, particularly adult epiglottitis, are more prevalent. Unusual microorganisms implicated in recent laryngeal abscess cases include *Mycobacterium tuberculosis* [19]*, Actinomyces odontolyticus* [20] and *Nocardia farcinia* [21], and. In our sample, the microorganisms most commonly associated with laryngeal abscesses were consistent with the predominant underlying pathology, particularly epiglottitis, with Streptococcus species being the most frequently identified pathogens. However, the bacterial diversity observed was highly variable.

**Fig. 1:**
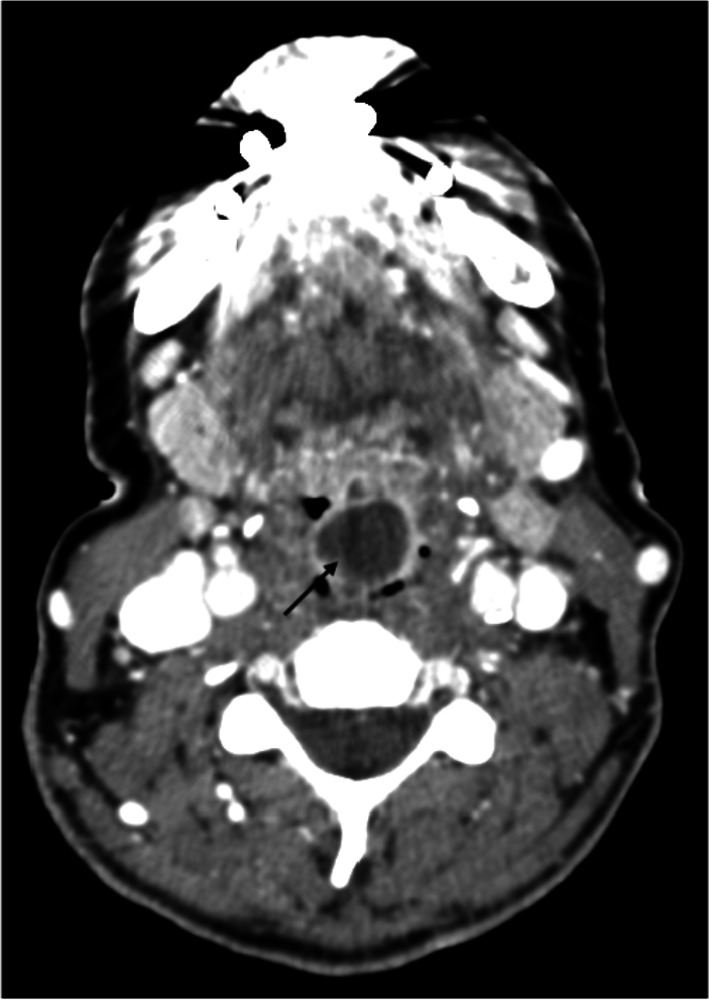
A large epiglottic abscessed collection (black arrow) with irregular contours that collapses the airway

Epiglottitis, once prevalent in children in industrialized countries before vaccination against Haemophilus influenzae (HI), remains a significant cause of laryngeal abscesses in adults, accounting for 61% in our series. Previous studies have reported epiglottitis complications leading to abscess formation in 12–19% of adult cases (Fig. 1) [9]. More than 60% of our patients with epiglottitis presented respiratory difficulty, with tracheostomy required in 2 cases and cricothyroidotomy in another, highlighting the potential for life-threatening complications with inadequate management. While abscess drainage typically necessitates general anesthesia and a transoral approach using various instruments, some studies have reported successful drainage using flexible endoscopy under local anesthesia [22].

**Fig. 2:**
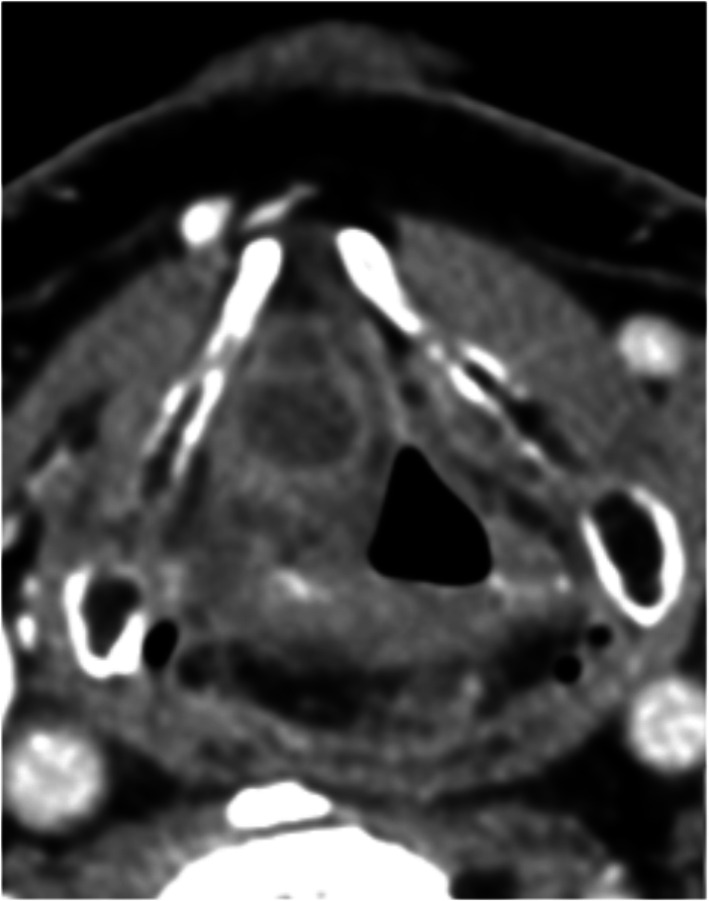
An internal laryngopyocele

**Fig. 3:**
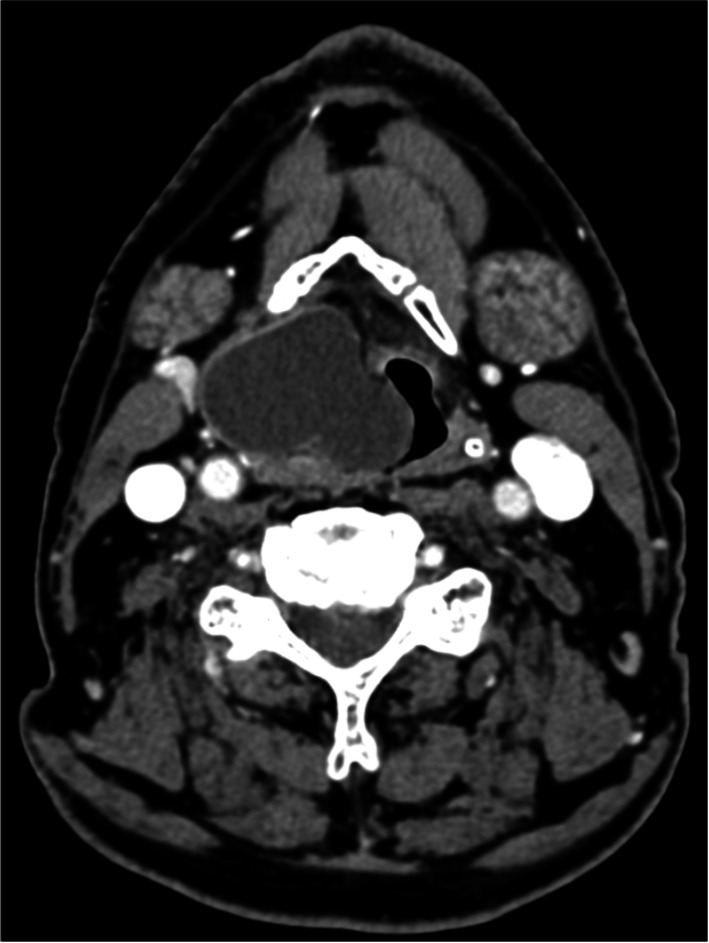
Laryngopyocele with external extension

Laryngopyoceles are laryngeal abscesses resulting from superinfection of a pre-existing laryngocele, characterized by pathological dilation of the laryngeal ventricular saccule, forming air-filled masses in the false vocal cord or neck (Figs. 2 and 3) [10]. These abscesses often present as acute laryngeal obstruction, requiring urgent interventions such as tracheostomy [10]. Treatment typically involves transoral endoscopic marsupialization for internal laryngoceles, while external/mixed laryngoceles may require more radical surgery following resolution of the infectious process [10].

Laryngeal cysts develop due to obstruction of mucinous gland drainage ducts, resulting in glandular secretion retention. While usually asymptomatic or presenting with mild symptoms, laryngeal cysts can undergo acute growth due to superinfection, potentially leading to airway obstruction and abscess formation.

Cricoid/retrocricoid abscesses are a particularly rare form of laryngeal abscesses, typically arising from mucosal injury leading to vascular damage and perichondritis. The formation of abscesses in this area is often triggered by factors such as foreign body impaction, external trauma, prolonged intubation, radiotherapy, or the placement of a nasogastric tube. While some cases may occur spontaneously, they are more commonly observed in immunosuppressed patients [8, 14].

Cricoid/retrocricoid abscesses are typically associated with unilateral or bilateral vocal cord paralysis due to compromised cricoarytenoid joint function [14]. Although our experience did not show any sequelae, these types of abscesses carry a high risk of morbidity due to the potential for late laryngeal stenosis [23]. Abscesses affecting the cricoarytenoid joint have also been reported in patients with conditions like rheumatoid arthritis, often presenting with vocal cord paralysis and edema upon laryngeal examination [11]. Some of these cases have been successfully managed with endoscopic drainage procedures [11].

Other types of laryngeal abscesses described in the literature, such as thyroid cartilage abscesses, involve inflammation of the laryngeal cartilaginous skeleton, known as perichondritis. This condition can lead to the formation of secondary abscesses between the inner and outer layers of the perichondrium. Typically, patients with thyroid cartilage abscesses present with symptoms such as dysphonia (hoarseness) and dyspnea (difficulty breathing) [5, 6].

The causal factors for thyroid cartilage abscesses are similar to those involved in cricoid cartilage abscesses and may also include spontaneous occurrences. Additionally, these abscesses may develop in the context of immunosuppressive conditions such as poorly controlled diabetes [5, 6].

In patients without an imminent risk of airway obstruction, the use of imaging tests plays a crucial role in decision-making and pre-surgical diagnosis. These tests help in identifying the exact location of the abscess and delineating the extent of the collection, aiding in treatment planning.

**Fig. 4:**
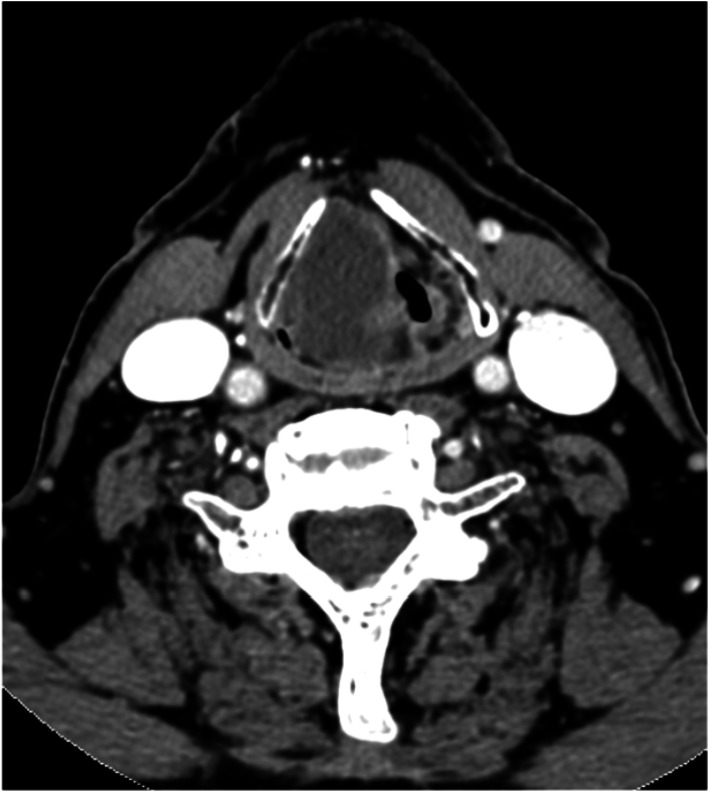
Large intralaryngeal abscess partially obstructing the airway

Among the various imaging modalities, computed tomography (CT) offers the highest diagnostic yield in the emergency department setting. Laryngeal abscesses typically appear on CT scans as an outer ring of hyperenhancement surrounding an inner image of hypoattenuation (Fig. 4). This characteristic imaging pattern helps in distinguishing abscesses from surrounding tissues.

In our series, presurgical contrast-enhanced CT scans were performed in 9 out of the 13 patients. This imaging approach allowed for accurate localization and characterization of the abscesses, facilitating surgical planning. However, in cases where the severity of the condition posed an immediate threat to the airway, interventions to stabilize the airway took precedence over imaging studies.

**Fig. 5:**
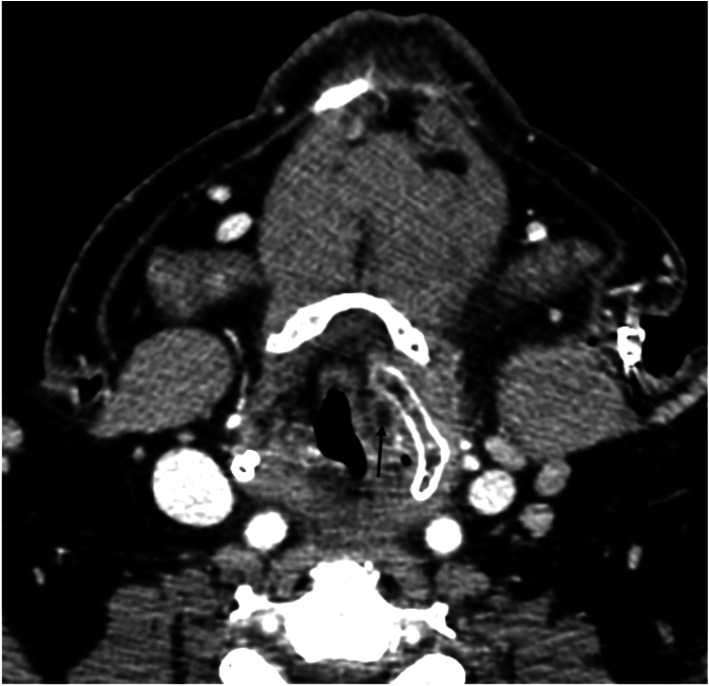
Intralaryngeal abscess localized in the left ventricular band (Black arrow)

**Fig. 6:**
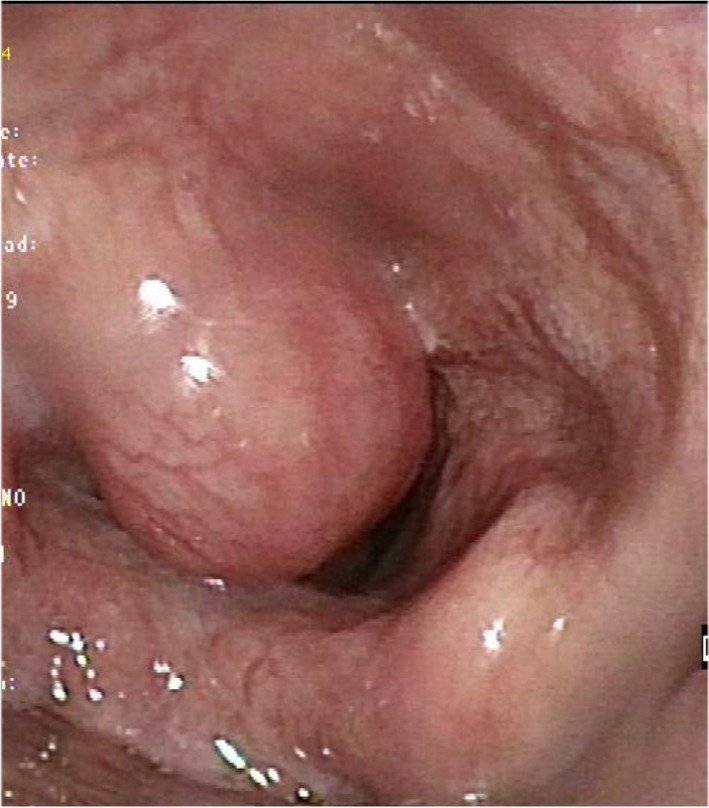
Videolaryngoscopy showing an internal laryngocele

Management of laryngeal abscesses requires prompt intervention and collaboration among a multidisciplinary team skilled in managing difficult airways. While small abscesses may respond well to antibiotic and corticosteroid treatment alone (Fig. 5) [16], larger abscesses typically require pus drainage, often performed under general anesthesia. Intubation with an endoscope or video laryngoscope may be necessary (Fig. 6), sometimes with the patient awake. Severe cases with airway obstruction may require a tracheostomy, as was necessary in five of our patients; in one case, a cricothyroidotomy was performed. Although all cases in our study were drained via a transoral route under general anesthesia, local anesthesia techniques using an endoscope have been described for the drainage of epiglottic abscesses [22].

Cultures should be obtained to guide antibiotic therapy adjustments. Initial treatment usually involves broad-spectrum intravenous antibiotics such as amoxicillin/clavulanic acid or third-generation cephalosporins, pending culture results. Systemic corticosteroids are commonly administered to alleviate laryngeal obstruction, although the scientific evidence supporting their efficacy is limited [24].

Fortunately, there were no fatalities in our series; however, two patients experienced serious systemic complications. While deaths due to laryngeal obstruction or severe neurological complications have been reported in the literature, our study did not observe such outcomes [25].

## Conclusions

Laryngeal abscesses are rare but potentially life-threatening conditions, with epiglottitis being the predominant cause. Prompt diagnosis, endoscopic evaluation, and appropriate management are crucial to prevent airway compromise and patient mortality. Surgical intervention, including tracheostomy, cricothyrotomy, or intubation using advanced techniques, may be necessary to secure an adequate airway. Favorable outcomes can be achieved with timely and comprehensive care.

## Data Availability

Data sharing not applicable to this article as no datasets were generated or analysed during the current study.
